# Forces and Moments Generated by Thermoforming Aligners and Direct-Printed Aligners: A Systematic Review

**DOI:** 10.3390/dj14090577

**Published:** 2026-09-08

**Authors:** Pritam Mohanty, Saundarya Priyadarshini, Debapreeti Mohanty, Margherita Tumedei, Massimo Del Fabbro, Parameswaran Tirunelveli Mani, Partha Pratim Chaudhary, Balaji Krishnan, Monalisa Das, Bhagabati Prasad Dash, Bhabani Shankar Biswal, Funda Goker, Saurav Panda

**Affiliations:** 1 Department of Orthodontics and Dentofacial Orthopaedics, Institute of Dental Sciences, Siksha ‘O’ Anusandhan University, Bhubaneswar 751029, India; pritammohanty@soa.ac.in (P.M.); monalisadas@soa.ac.in (M.D.); bhabanishankarbiswal@soa.ac.in (B.S.B.); 2 Department of Paediatric Surgery, All India Institute of Medical Sciences, New Delhi 110029, India; priyadarshini.saundarya@gmail.com; 3 Department of Conservative Dentistry and Endodontics, Institute of Dental Sciences, Siksha ‘O’ Anusandhan University, Bhubaneswar 751029, India; debapreetimohanty@soa.ac.in; 4 Department of Biomedical, Surgical and Dental Sciences, Universitàdegli Studi di Milano, 20122 Milan, Italy; massimo.delfabbro@unimi.it (M.D.F.); fundagoker@aydin.edu.tr (F.G.); 5 Fondazione IRCCS Ca’Granda Ospedale Maggiore Policlinico, 20122 Milan, Italy; 6 Department of Orthodontics, Tagore Dental College and Hospital, Rathinamangalam 600127, India; drparameswaran@tagoredch.in (P.T.M.); balajikrishnan@tagoredch.in (B.K.); 7 Department of Orthodontics, R. Ahmed Dental College & Hospital, Kolkata 700014, India; doctorpartha@gmail.com; 8 Department of Orthodontics and Dentofacial Orthopaedics, Kalinga Institute of Dental Sciences, Bhubaneswar 751024, India; bhagabati.dash@kids.ac.in; 9 Department of Oral and Maxillofacial Surgery, Faculty of Dentistry, Istanbul Aydın University, 34000 Istanbul, Turkey; 10 Department of Periodontics, Institute of Dental Sciences, Siksha ‘O’ Anusandhan University, Bhubaneswar 751029, India

**Keywords:** thermoforming aligners, direct-printed aligners, orthodontics, force retention, biomechanical properties, tooth movement, clear aligners

## Abstract

**Objectives:** This systematic review aims to compare the forces and moments generated by thermoforming aligners and direct-printed aligners in guiding tooth movement in orthodontic patients, evaluating their impact on orthodontic outcomes. **Methods:** A systematic search was conducted in PubMed, Web of Science, and Scopus databases, covering studies on thermoforming and direct-printed aligners from inception to October 2024. Eligible studies included randomized controlled trials, quasi-experimental studies, and observational studies assessing forces and moments generated by these aligners on teeth. In vitro studies pertinent to the topic were also considered. Data extraction focused on study characteristics, aligner details, force and moment measurements, and clinical outcomes. Quality assessment utilized the ROBINS- I for Risk of Bias. The review has been registered on PROSPERO CRD42024620341. **Results:** A total of nine studies met the inclusion criteria. Findings indicate that thermoforming aligners, while effective in initial force application, exhibit significant force decay over time due to material stress relaxation. In contrast, direct-printed aligners maintain more consistent force levels, potentially resulting in more predictable and sustained tooth movement. Both aligner types vary in their ability to control force direction and magnitude, with direct-printed aligners showing advantages in customization and force retention. **Conclusions:** The biomechanical properties of thermoforming and direct-printed aligners offer distinct advantages and limitations. Direct-printed aligners may enhance clinical predictability and reduce treatment time through consistent force application, though further research is necessary to validate these benefits in clinical settings. This review underscores the importance of material selection and fabrication method in optimizing orthodontic outcomes.

## 1. Introduction

Thermoforming aligners and direct-printed aligners are gaining significant attention within the field of orthodontics due to their growing potential for customizing dental movements with precision and comfort. This systematic review explores the forces and moments generated by these two types of clear aligners, examining their effectiveness and mechanics in guiding tooth movement. The biomechanical forces produced by thermoforming and direct-printed aligners are critical in shaping the path and rate of tooth movement, making an understanding of these forces essential for optimal clinical outcomes.

Thermoforming and direct-printed aligners are both components of a growing trend towards non-invasive, patient-centered orthodontic treatments that prioritize esthetics, comfort, and efficient treatment times. Traditionally, orthodontic treatment relied on metal braces that, while effective, lacked in terms of patient comfort and visual appeal. Clear aligners, however, have addressed these concerns, and both thermoforming and 3D printing technologies have been instrumental in advancing aligner design and functionality. Although similar in appearance, thermoformed and direct-printed aligners differ significantly in their material characteristics, production processes, and force application mechanisms, each of which directly impacts clinical effectiveness [1,2,3,4,5,6,7,8,9,10].

The literature emphasizes that both types of aligners—thermoforming and direct-printed—have unique mechanical properties that influence their ability to generate the necessary forces and moments for effective orthodontic treatment. However, inconsistencies remain regarding the magnitude and duration of forces generated, as well as how these forces translate to clinical outcomes. Some studies report that thermoforming aligners may exhibit rapid force decay, resulting in suboptimal force application over time, while direct-printed aligners may demonstrate enhanced force retention due to improved material properties [11]. Furthermore, the direct-printing process may facilitate adjustments in force application tailored to specific tooth movements, potentially enhancing treatment efficiency and reducing the duration of orthodontic therapy.

Recent evidence syntheses further emphasize that the validity of planned tooth movements with clear aligners is movement-specific rather than uniform. An overview of systematic reviews and meta-analyses reported variable predictability of tooth movements, particularly for canine rotations [12]. A 2024 systematic review likewise concluded that clear aligners can effectively control several orthodontic movements, although limitations remain for more complex movements [13]. More movement-specific evidence has also shown that distalization can be achieved with clear aligners, while the predictability of lower incisor intrusion remains variable [14,15]. A recent contemporary review categorized extrusion, anterior intrusion, canine/premolar rotation, and torque among the less predictable movements, whereas expansion and maxillary buccal-segment distalization showed higher predictability [16]. A further 2025 systematic review highlighted the influence of movement type, treatment planning, case complexity, and patient compliance on aligner treatment predictability [17]. These findings indicate that clinical tooth-movement validity should be interpreted in a movement-specific context and that the biomechanical force and moment characteristics evaluated in the present review should not be considered synonymous with clinical movement predictability.

One of the emerging focuses in orthodontic research is the comparison of force retention between these two aligner types. Force retention, or the aligner’s ability to sustain force over time without degradation, is essential for maintaining consistent tooth movement throughout the treatment period [18]. Studies have shown that thermoformed aligners experience significant force decay within the first 24 to 48 h, which may require the patient to change aligners more frequently or undergo adjustments [19,20,21]. Direct-printed aligners, however, tend to exhibit a more sustained force profile due to enhanced material durability and customization potential, suggesting that they may offer a more consistent force application throughout each wear cycle [22].

Furthermore, direct-printing technologies allow for innovative material developments aimed at improving the biomechanical properties of aligners. For instance, recent research has explored flexible and rigid polymers in combination, creating multi-material aligners that can apply both light and strong forces depending on the specific movement needed for each tooth [22,23]. The ability to tailor material properties in this way can optimize the balance of comfort and force effectiveness, potentially leading to shorter treatment times and improved patient outcomes.

A systematic review of the forces and moments generated by thermoforming and direct-printed aligners is essential to elucidate these differences and inform clinical decision-making in orthodontics. This review will assess and compare existing evidence on the biomechanics of thermoforming and direct-printed aligners, exploring how fabrication methods impact force generation, retention, and decay, and evaluating their influence on orthodontic outcomes. By synthesizing findings from recent studies, this review aims to provide clinicians with an in-depth understanding of the biomechanical implications of each aligner type, ultimately guiding the selection of the most effective aligner fabrication method for individual patient needs.

## 2. Materials and Methods

This systematic review and meta-analysis aimed to evaluate the forces and moments generated by thermoforming aligners and direct-printed aligners in orthodontic treatment, following PRISMA guidelines (Table S1). The review has been registered on PROSPERO CRD42024620341.

Population (P):Patients undergoing orthodontic treatment with aligners (for clinical studies); or standardized dental models and typodont systems (for in vitro and finite element studies).

Intervention (I): Thermoformed aligners.

Comparison (C): Direct-printed aligners.

Outcome (O): Forces and moments generated by the aligners, including magnitude, consistency, and clinical efficacy.

Study Design (S): Laboratory studies: in vitro studies, finite element analyses, and experimental biomechanical studies. Clinical studies: randomized controlled trials, quasi-experimental, observational, or comparative trials assessing forces and moments generated by aligners in patients.

Review Questions

What are the differences in forces and moments generated by thermoformed aligners compared to direct-printed aligners?

### 2.1. Eligibility Criteria

We included randomized controlled trials (RCTs), quasi-experimental studies, observational studies and in vitro studies that investigated the forces and moments generated by thermoformed aligners or direct-printed aligners on teeth. Studies with participants of any age undergoing orthodontic treatment with aligners were included. The interventions of interest were thermoforming aligners and direct-printed aligners, compared to each other or to other aligner types and methods. The primary outcomes were measurements of forces (measured in Newtons) and moments (measured in Newton-millimeters) exerted on teeth. There were no restrictions on publication date or study setting. Studies published in languages other than English were excluded.

### 2.2. Information Sources

A comprehensive search of electronic databases was conducted, including PubMed, Web of Science, and Scopus, covering the period from inception to October 2024. Snowball referencing was done for the existing literature.

### 2.3. Search Strategy

The search strategy was developed using terms related to aligner forces and moments in orthodontics: (“Thermoforming aligners” OR “direct-printed aligners”) AND (“forces” OR “moments” OR “orthodontics” OR “aligner mechanics”)

This strategy was adapted as needed for each database to ensure a comprehensive search. For records management, all search results were exported into a reference management software (Zotero) to facilitate organization and analysis. Duplicate records, identified during the import and organization process in the citation manager, were systematically removed to ensure that each entry was unique.

### 2.4. Study Selection

**Figure 1:**
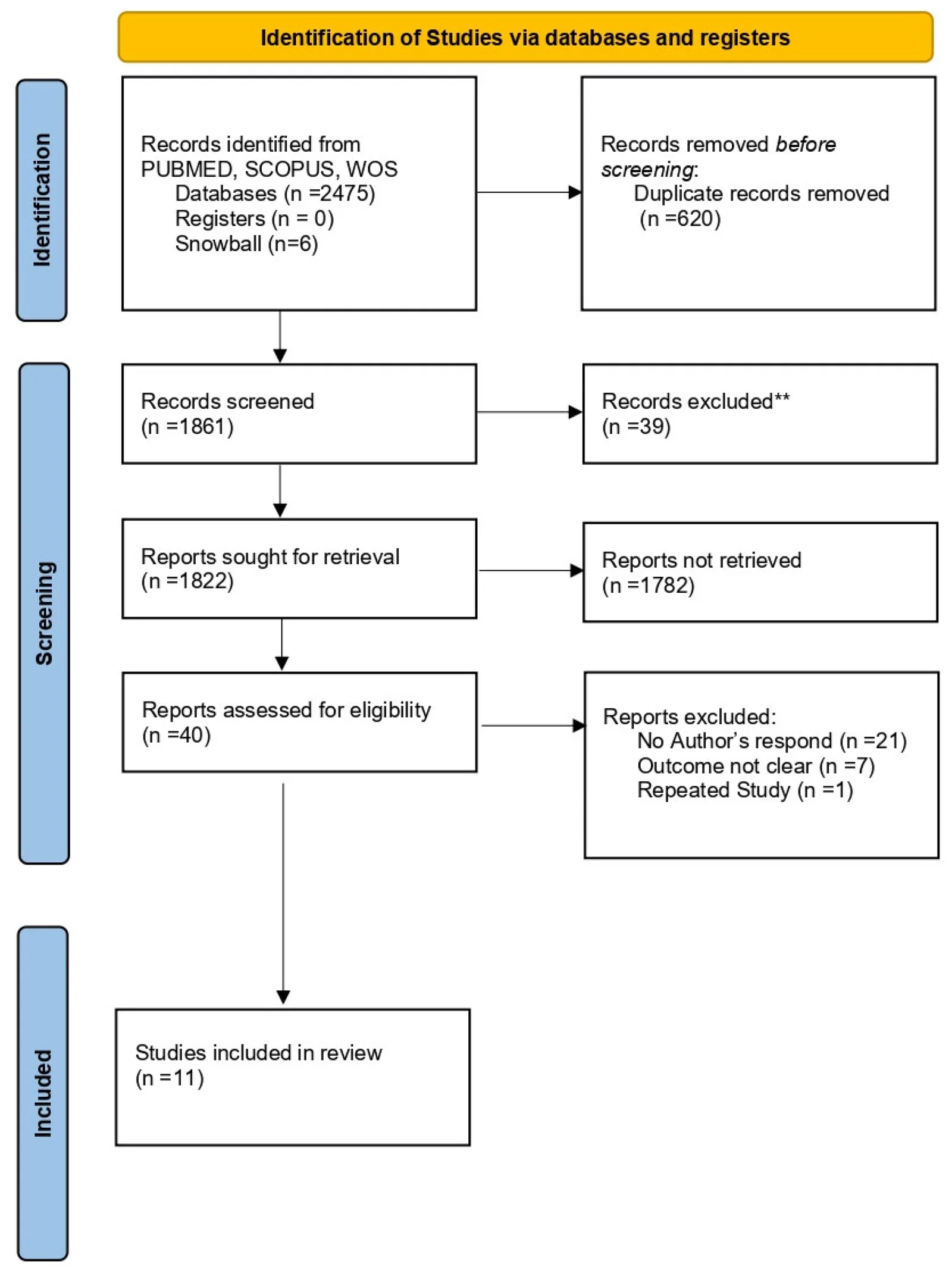
PRISMA flow chart diagram.

Two independent reviewers (P.M., So.P.) screened the titles and abstracts of identified studies for potential inclusion. Full texts of potentially eligible studies were then assessed based on the eligibility criteria. Disagreements were resolved through discussion or with input from a third reviewer. The selection process was documented and illustrated using a PRISMA flow diagram (Figure 1).

### 2.5. Data Collection Process

Data extraction was conducted independently by two reviewers (S.P., P.M.) using a standardized data collection form in Microsoft Excel. Extracted data included study characteristics (authors, publication year, country), participant characteristics (age, sex, orthodontic condition), aligner type (thermoforming or direct printed), aligner details (material, thickness, method of fabrication), force and moment measurement details (type of measurement, tools used, tooth positions), and results.

### 2.6. Risk of Bias Assessment

Risk of bias of the included studies was assessed using the ROBINS-I (Risk Of Bias In Non-randomized Studies—of Interventions) tool, which considers:

Bias due to confounding.

Bias due to selection of participants.

Bias in the classification of interventions.

Bias due to deviations from intended interventions.

Bias in the measurement of outcomes.

Missing outcome data.

Selection of the reported result.

Risk of bias for in vitro studies was assessed using the QUIN tool [23]. This tool was specifically developed for in vitro studies in dentistry and consists of 12 criteria, each scored as adequately specified (2 points), inadequately specified (1 point), not specified (0 points), and not applicable (criteria excluded from final calculation). The scores thus assessed were used to classify the in vitro study as high, medium, or low risk (>70%: low risk of bias, 50% to 70%: medium risk of bias, and <50%: high risk of bias) by using the following formula: Final score = (Total score × 100)/(2 × number of criteria applicable).

## 3. Results

Given the differences in protocols, study design, aims and materials among the different studies, it was not possible to perform any meta-analysis to aggregate findings, and the results are presented in a narrative way.

### 3.1. Study Characteristics

A total of 11 studies [24,25,26,27,28,29,30,31,32,33,34] were included, spanning six in vitro and finite element studies [24,25,26,27,28,29,30] and three experimental studies [25,31,32,33,34] focused on the biomechanical properties, patient compliance, and clinical efficacy of thermoformed aligners. The studies examined different aligner materials (such as PET-G, Biolon) and thicknesses (ranging from 0.3 mm to 1 mm), primarily analyzing outcomes like force generation, moments applied, and stress relaxation. The main features of the included studies are summarized in Table 1.

### 3.2. Risk of Bias Assessment

**Figure 2:**
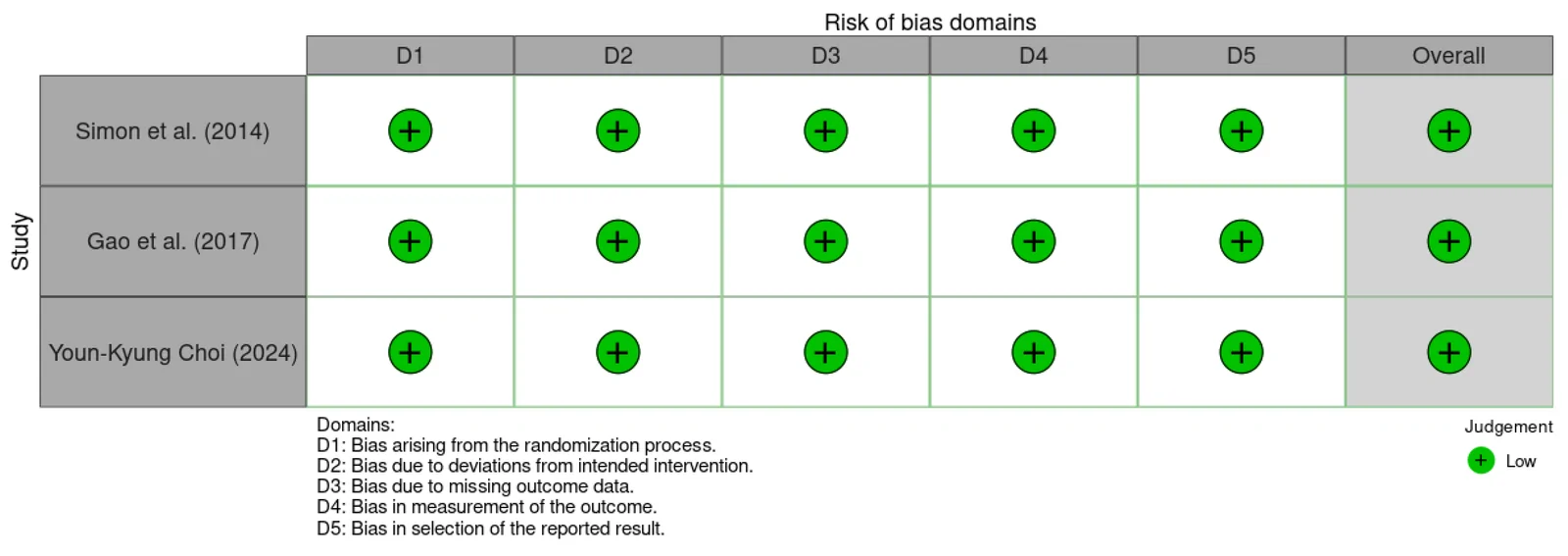
Risk of bias for included experimental studies [25,31,32].

Table 2 and Figure 2 summarize the results of the risk of bias assessment. All included in vitro studies (Table 2) were assessed using the QUIN tool and demonstrated a medium risk of bias. The three experimental studies [25,31,32] were evaluated using the ROBINS-I tool (Figure 2) and were judged to have a low risk of bias.

### 3.3. Biomechanical Properties of Thermoformed Aligners

Force magnitude and distribution are central to the clinical efficacy of aligners, as emphasized by Hou et al. [36] and Shirey et al. [35], who measured forces required for effective tooth movement. Several studies, including Cremonini et al. [37] and Dalaie et al. [38], reported that aligner thickness did not significantly impact force magnitude, with aligners of 0.5 mm, 0.625 mm, and 0.75 mm exerting similar forces for tooth movements such as tipping and rotation. Dalaie et al. [38] noted a decline in force over time, suggesting that stress relaxation affects aligner efficacy as wear duration increases. Lego et al. [34] reported that 3D-printed devices are characterized by increased absorbance, decreased transmittance, and more color change compared with the thermoformed devices. On the other hand, Cengiz et al. [33] reported a significant difference in terms of clinical efficacy and patient satisfaction. The aligners with a 0.5 mmthickness showed enhanced comfort for subjects, but a 0.75 mm-thick aligner demonstrated a more efficient clinical outcome.

Studies that quantified moment-to-force ratios, such as those by Wang et al. [39], highlighted the stability of moment-to-force (M/F) ratios across different thicknesses, indicating that thickness alone may not substantially influence biomechanical performance. This was consistent with findings from Iliadi et al. [40], who also concluded that thickness had minimal impact on M/F ratios, specifically for palatal tipping movements.

Multiple studies observed a reduction in force over time due to stress relaxation. Cremonini et al. [41] documented force decay rates, noting a significant drop within the first two weeks of wear. This suggests that regular replacement of aligners may be necessary to maintain effective force levels throughout treatment.

### 3.4. Clinical Efficacy and Treatment Outcomes

Clinical studies reported moderate success rates in achieving alignment targets, with Timm et al. [42] finding that most aligners could maintain accuracy when used according to recommended replacement intervals. However, differences in material and thickness did not consistently translate to improved clinical outcomes. Regalado-Bazan CF et al. [43] observed that retention was more successful in patients who adhered to a structured post-treatment protocol involving retainers. This highlights the importance of continued retention to prevent relapse after active treatment, a finding consistent across several included studies.

### 3.5. Patient Compliance and Satisfaction

Studies focusing on patient compliance, such as Alansari RA et al. [44], reported that esthetic satisfaction was a major motivator, with patients showing higher wear-time adherence compared to traditional braces. However, Robertson L et al. [45] noted variability in self-reported compliance, suggesting that self-reports may overestimate true compliance rates. Esthetic satisfaction: patients preferred thermoformed aligners for their esthetic appeal, with satisfaction ratings consistently high across studies, especially in younger adults. These findings underscore the advantage of clear aligners for patients concerned with appearance during orthodontic treatment.

### 3.6. Long-Term Effects

Several studies addressed the long-term effects of thermoformed aligners, emphasizing the necessity of retainer use to maintain alignment. Regalado-Bazan et al. [43] and Kramer et al. [46] both reported relapse in patients who did not follow retainer protocols, indicating that sustained benefits of aligners are closely tied to post-treatment adherence.

## 4. Discussion

The evaluation of thermoformed aligners spans multiple facets, including material durability, clinical efficacy, patient compliance, esthetic appeal, and long-term outcomes. Studies by various authors [43,44,45,46] provide insights into these areas, each contributing to a nuanced understanding of the benefits and challenges associated with thermoformed aligners.

### 4.1. Material Properties and Mechanical Performance

The mechanical properties of thermoformed aligners, such as stress resistance and force retention, were investigated by several studies. For instance, Cremonini et al. [41] analyzed the effect of thermo-mechanical aging on aligners and reported a reduction in the force exerted over time, suggesting that repeated thermal cycles could compromise the aligner’s ability to maintain optimal tooth-movement forces. Similarly, Dalaie et al. [38] focused on the material’s durability under repeated forces, indicating a decline in stress resistance with extended usage. In contrast, Lombardo et al. [18] highlighted the advantages of PET-G (polyethylene terephthalate glycol) in achieving a balance between flexibility and durability, demonstrating that material choice substantially impacts aligner performance. These findings collectively underscore the need for further research into materials that can withstand prolonged use without a loss in force retention. Improved material properties could potentially enhance treatment longevity and reduce the frequency of aligner replacements.

### 4.2. Outcome Measures and Clinical Efficacy

Force magnitude and distribution are central to the clinical efficacy of aligners, as emphasized by Shirey et al. [35], who measured forces required for effective tooth movement. They found that aligners with higher force levels were generally more effective for complex orthodontic cases, though this often came at the expense of patient comfort. Similarly, Elshazly et al. [10] quantified the forces and moments delivered by aligners using advanced 3D modeling and observed that precise force calibration improved treatment outcomes but highlighted potential discomfort for patients with higher force aligners.

Conversely, Cremonini F et al. [37] raised concerns about stress relaxation, wherein force levels diminished over time, potentially compromising treatment progress. Collectively, these studies illustrate the challenge of balancing force efficacy and comfort, which is vital for achieving desired clinical outcomes without negatively impacting patient experience.

### 4.3. Patient Compliance and Esthetic Satisfaction

Esthetic satisfaction plays a significant role in patient compliance, as highlighted by Schafer K et al. [38], who found higher compliance rates among aligner users compared to those using traditional braces. Their study showed that the discrete appearance of aligners encouraged patients, particularly young adults, to adhere more consistently to the treatment schedule. This finding was supported by findings from Alansari et al. [44], who studied compliance across age groups and observed a positive correlation between esthetic satisfaction and daily wear duration.

However, Robertson L et al. [45] questioned the reliability of self-reported compliance data, suggesting that esthetic satisfaction alone might not guarantee consistent aligner use. This emphasizes the importance of regular follow-up appointments to monitor compliance and address any challenges patients may face during treatment.

### 4.4. Long-Term Stability and Retention After Aligner Treatment

Long-term efficacy and stability of alignment achieved through thermoformed aligners were assessed in studies like that of Regalado-Bazan et al. [43], who documented alignment stability over a five-year period, noting higher relapse rates in patients who did not continue using retainers post-treatment. However, Kramer et al. [46] found that patients who adhered to a periodic retainer routine post-treatment maintained better alignment stability, suggesting that sustained benefits of aligners are closely tied to compliance with retention protocols. The findings of this review align with those reported by Iliadi et al. (2019) [40], who systematically analyzed forces and moments generated by different aligner types. Similar to our observations, Iliadi et al. noted that aligner thickness, particularly for PET-G materials, did not significantly alter the moment-to-force (M/F) ratio, which remained consistent across thicknesses of 0.5 mm, 0.625 mm, and 0.75 mm for specific tooth movements, such as palatal tipping of the upper central incisor [40]. Both reviews emphasize that while thickness can influence rigidity, it does not necessarily correlate with an increase in effective delivery force, which is a critical factor for clinical implications. On the contrary, Cengiz et al. reported an increased efficacy of 0.75mm aligners but a decrease in patient satisfaction levels in a randomized clinical trial on 24 subjects [33]. Additionally, Iliadi et al.’s focus on laboratory-based studies underscores the necessity for in vivo research to validate the clinical relevance of these biomechanical findings, a sentiment also reflected in our review’s conclusions regarding the limitations of lab-only data.

A meta-analysis could not be conducted in this review due to significant heterogeneity across the included studies. Variability in study design, materials, aligner thicknesses, measurement techniques, and experimental settings made it difficult to combine data in a meaningful way. Many studies used different models and measurement tools to assess forces and moments, leading to inconsistent results that are not directly comparable. Additionally, the lack of standardization in aligner properties (such as type, thickness, and specific orthodontic movements) further compounded these differences, resulting in insufficient common data points to perform a robust statistical synthesis. This variability underscores the need for standardized protocols in future research to enable more reliable comparisons and potential meta-analyses.

Importantly, these observations should be considered alongside recent clinical evidence on aligner tooth-movement predictability. Current evidence syntheses indicate that the expression of planned movement varies substantially by movement type, with rotation, intrusion, extrusion, and torque generally less predictable than selected distalization and expansion movements [12,13,14,15,16,17]. Thus, the force and moment findings reported here provide a biomechanical basis for understanding aligner performance but should be interpreted cautiously when extrapolating to clinical tooth-movement accuracy. The findings point to the critical role of retention in maintaining long-term outcomes, indicating that successful treatment with thermoformed aligners requires a commitment to post-treatment protocols to minimize relapse.

### 4.5. Limitations and Future Directions

A common limitation among the reviewed studies is the variability in methodology and evaluation criteria, making direct comparisons challenging. Standardized protocols are essential for future research to improve the comparability of findings across studies. Additionally, advancements in materials science and emerging technologies, such as 4D printing, may allow for the development of aligners with adaptive properties that better meet patient needs over time.

## 5. Conclusions

This systematic review synthesized evidence from nine in vitro, finite element, and experimental studies comparing the biomechanical properties of thermoforming and direct-printed clear aligners. Thermoforming aligners fabricated from PET-G materials demonstrated significant force decay—particularly within the first 24 to 48 h of wear—attributable to stress relaxation of the thermoplastic polymer under repeated loading. Aligner thickness (0.5–0.75 mm) did not substantially alter moment-to-force ratios for specific tooth movements such as palatal tipping, suggesting that material choice outweighs thickness in determining biomechanical performance. Direct-printed aligners produced via SLA or DLP technologies demonstrated more sustained force profiles and greater dimensional accuracy, offering enhanced customization of force magnitude and direction. However, the currently available evidence is predominantly laboratory-based; clinical validation remains limited, and no meta-analysis was feasible owing to significant heterogeneity in study design, materials, and measurement protocols. Future research should prioritize standardized in vivo studies, longer wear-period assessments, and head-to-head clinical trials comparing thermoforming and direct-printed aligners across different tooth-movement types to establish definitive clinical guidelines.

## Figures and Tables

**Table 1:** Characteristics of included studies.

Title	Year	Author	Sample Size	Country	Study Design	Outcome Measured
The effectiveness of orthodontic treatment with clear aligners in different thicknesses	2025	Cengiz et al. [33]	24	Turkey	Randomized clinical trial	Evaluate the efficacyof different thicknesses of clear aligners.
Comparison of optical properties and color stability of 3-dimensional (3D) printed shape memory and thermoformed clear aligners: A single-center, prospective clinical trial study	2025	Lego et al. (2025) [34]	24	India	Prospective clinical trial	Compare the optical properties and color stability of direct 3D-printed and thermoformed clear aligners after intraoral wear.
Effect of material composition and thickness of orthodontic aligners on the transmission and distribution of forces: an in vitro study	2024	Elshazly et al. (2024) [30]	60	Egypt	In vitro study	Force values (in Newtons) at the incisal, middle, and cervical thirds of the facial surface of the maxillary central incisor, as well as the overall force over the entire tooth surface. The net active force between the incisal and cervical thirds (F I -F C) and between the incisal and middle thirds (F I -F M) to investigate the effect of material and thickness on the moment of force and the resulting type of tooth movement.
Analysis of the Forces and Moments in Canine Bodily Movement with Different Clear Aligners’ Extraction Space Designs	2024	Youn-Kyung Choi (2024) [31]	120	South Korea	Experimental study	The primary outcomes measured in this study were the forces (buccolingual force (Fx), mesiodistal force (Fy), and intrusive force (Fz)) and moments (angulation moment (Mx), inclination moment (My), and rotation moment (Mz)) generated on the maxillary right canine during its 0.25 mm distal bodily movement with different clear aligner (CA) designs in the edentulous region.
Forces and moments generated by 3D direct-printed clear aligners of varying labial and lingual thicknesses during lingual movement of maxillary central incisor: an in vitro study	2023	Grant et al. (2023) [24]	50	United States	In vitro study	The primary outcomes measured in this study were the forces and moments generated by direct 3D-printed clear aligners (DPAs) during lingual movement of a maxillary central incisor, measured in Newtons (N) and Newton-millimeters (N·mm), respectively.
Forces and moments generated during extrusion of a maxillary central incisor with clear aligners: an in vitro study	2023	Mckay et al. (2023) [28]	90	South Korea	In vitro study	The primary outcomes measured in this study are the forces and moments experienced by the maxillary central incisors (UL1 and UR1) during extrusion, measured in Newtons (N) for forces and Newton-millimeters (Nmm) for moments.
Torque movement of the upper anterior teeth using a clear aligner in cases of extraction: a finite element study	2022	Cheng et al. (2022) [27]	Not mentioned (the paper does not explicitly state the sample size, but it appears to have used a single participant to create the finite element model)	Not mentioned (the country of the authors is not mentioned in the paper)	Finite element analysis (FEA) study	Rotation angle of the upper central incisor in the sagittal direction. Location of the rotation center of the upper central incisor. Highest vonMises stress values for the upper central incisor root, periodontal ligament, and alveolar bone.
Distributed Force Measurement and Mapping Using Pressure-Sensitive Film and Image Processing for Active and Passive Aligners on Orthodontic Attachments	2022	Zamani et al. (2022) [29]	6 aligners total—3 passive and 3 active	Malaysia	In vitro study	The primary outcome measured in this paper is the topographical force mapping and the range of forces exerted by clear aligners on orthodontic attachments, with the mean force ranging from 6.2 to 6.3 N for active attachments and 4.8–4.9 N for passive attachments, resulting in an average net force of 1.3–1.4 N.
Force changes associated with differential activation of en-masse retraction and/or intrusion with clear aligners	2021	Zhu et al. (2021) [26]	60	China	In vitro study	The primary outcome measured in this study was the three-dimensional (3D) forces created by clear aligners on mandibular teeth during differential activation with en-masse retraction and/or intrusion.
Forces and moments delivered by the PET-G aligner to a maxillary central incisor for palatal tipping and intrusion	2017	Gao et al. (2017) [32]	54	Germany	Experimental study	The primary outcomes measured in this study were the forces (F x, F y, F z ) and moments (M x, M y, M z ) delivered by PET-G aligners to a maxillary central incisor during palatal tipping and intrusion.
Forces and moments generated by removable thermoplastic aligners: Incisor torque, premolar derotation, and molar distalization	2014	Simon et al. (2014) [25]	30	Germany	Experimental study	(1) Forces and moments (in all 3 spatial dimensions) delivered by individual Invisalign aligners. (2) Forces and moments (in all 3 spatial dimensions) delivered by a series of Invisalign aligners. (3) The influence of attachments and power ridges on the force transfer from Invisalign aligners.

**Table 2:** QUIN scores for invitro studies.

Ref. N.	Author, Year	1 Clearly Stated Aims/Objectives	2 Detailed Explanation of Sample Size Calculation	3 Detailed Explanation of Sampling Technique	4 Details of Comparison Group	5 Detailed Explanation of Methodology	6 Operator Details	7 Randomization	8 Method of Measurement of Outcome	9 Outcome Assessor Details	10 Blinding	11 Statistical Analysis	12 Presentation of Results	Overall Score (% Tot)
[33]	Cengiz et al. (2025)	2	1	0	2	2	0	1	2	2	0	2	2	16 (66.6)
[34]	Lego et al. (2025)	2	1	0	2	2	1	0	2	2	0	2	2	16 (66.6)
[24]	Grant et al. (2023)	2	1	0	2	2	0	1	2	2	0	2	2	16 (66.6)
[26]	Zhu et al. (2021)	2	1	0	2	2	1	0	2	2	0	2	2	16 (66.6)
[27]	Cheng et al. (2022)	2	1	0	2	2	1	0	2	2	0	2	2	16 (66.6)
[28]	Mckay et al. (2023)	2	1	0	2	2	1	0	2	2	0	2	2	16 (66.6)
[35]	Shirey et al. (2023)	2	1	0	2	2	1	0	2	2	0	2	2	16 (66.6)
[30]	Elshazly et al. (2024)	2	1	0	2	2	1	0	2	2	0	2	2	16 (66.6)

Table 2 RoB of the included studies.

## Data Availability

The original contributions presented in this study are included in the article and Supplementary Materials. Further inquiries can be directed to the corresponding authors.

## Supplementary Materials

The following supporting information can be downloaded at: https://www.mdpi.com/article/10.3390/dj14090577/s1. Table S1: PRISMA 2020 checklist.
